# The Ribbon-Helix-Helix Domain Protein CdrS Regulates the Tubulin Homolog *ftsZ2* To Control Cell Division in Archaea

**DOI:** 10.1128/mBio.01007-20

**Published:** 2020-08-11

**Authors:** Cynthia L. Darnell, Jenny Zheng, Sean Wilson, Ryan M. Bertoli, Alexandre W. Bisson-Filho, Ethan C. Garner, Amy K. Schmid

**Affiliations:** a Biology Department, Duke University, Durham, North Carolina, USA; b Department of Molecular and Cellular Biology, Harvard University, Cambridge, Massachusetts, USA; c Department of Biology, Rosenstiel Basic Medical Science Research Center, Brandeis University, Waltham, Massachusetts, USA; d Center for Genomics and Computational Biology, Duke University, Durham, North Carolina, USA; Max Planck Institute for Terrestrial Microbiology

**Keywords:** archaea, cell division, gene regulation, transcription factors, video microscopy

## Abstract

Precise control of the cell cycle is central to the physiology of all cells. In prior work we demonstrated that archaeal cells maintain a constant size; however, the regulatory mechanisms underlying the cell cycle remain unexplored in this domain of life. Here, we use genetics, functional genomics, and quantitative imaging to identify and characterize the novel CdrSL gene regulatory network in a model species of archaea. We demonstrate the central role of these ribbon-helix-helix family transcription factors in the regulation of cell division through specific transcriptional control of the gene encoding FtsZ2, a putative tubulin homolog. Using time-lapse fluorescence microscopy in live cells cultivated in microfluidics devices, we further demonstrate that FtsZ2 is required for cell division but not elongation. The *cdrS-ftsZ2* locus is highly conserved throughout the archaeal domain, and the central function of CdrS in regulating cell division is conserved across hypersaline adapted archaea. We propose that the CdrSL-FtsZ2 transcriptional network coordinates cell division timing with cell growth in archaea.

## Abstract (precis)

Healthy cell growth and division are critical for individual organism survival and species long-term viability. However, it remains unknown how cells of the domain *Archaea* maintain a healthy cell cycle. Understanding the archaeal cell cycle is of paramount evolutionary importance given that an archaeal cell was the host of the endosymbiotic event that gave rise to eukaryotes. Here, we identify and characterize novel molecular players needed for regulating cell division in archaea. These molecules dictate the timing of cell septation but are dispensable for growth between divisions. Timing is accomplished through transcriptional control of the cell division ring. Our results shed light on mechanisms underlying the archaeal cell cycle, which has thus far remained elusive.

## INTRODUCTION

The cell cycle proceeds through an ordered progression of molecular events, including cell volume increase, DNA replication, segregation, and cytokinesis. The fine-tuned control between these processes has been studied intensely for decades, yielding deep insight into cell cycle mechanisms. To date, such work has focused on bacterial and eukaryotic model organisms. In contrast, the archaeal cell cycle remains virtually unexplored despite its importance as the evolutionary progenitor of eukaryotes (1). The few studies that have been conducted on the cell cycle in archaeal model organisms point to a hybrid of eukaryotic and bacterial features with differential assortment of these features throughout the archaeal lineages. For example, in *Crenarchaeota*, the cell cycle phases, molecular machinery of DNA replication, and cell division are largely conserved with eukaryotes (2–5). In contrast, the cell cycle in the lineage Euryarchaeota retains features of all three domains, including a bacterial FtsZ system of cell division (6), a eukaryotic system for DNA replication (5), and archaea-specific firing of replication origins (7).

The tubulin homolog FtsZ has been studied in detail in many bacteria for its central role in cell division. In most bacteria, FtsZ monomers assemble into short filaments that form the cytokinetic ring at midcell, which constricts to divide the mother cell into two daughters of equal size (8–12). FtsZ in archaea appears to function similarly, as previous fluorescence imaging experiments in fixed (13–16) and live (17–19) hypersaline adapted archaeal cells (species Haloferax volcanii [Hfx. volcanii]) demonstrated Z-like rings forming at midcell. However, all known halophilic archaeal genomes encode multiple tubulin-like proteins (13, 20), so the function and mechanism of these proteins in cell division remain unclear.

Halobacteria, a clade of hypersaline-adapted Euryarchaeota, provide excellent model systems for understanding cell cycle mechanisms and how they are regulated. In particular, for the model species Halobacterium salinarum (Hbt. salinarum), Hfx. volcanii, and Hfx. mediterranei, large and facile toolkits enable genetic manipulation (knockouts, overexpression, etc.) (21–24). For *Hbt. salinarum* strain NRC-1, large systems biology data sets, including transcriptomic profiles under a wide array of growth and stress conditions, enable rapid hypothesis generation regarding gene functions (25, 26). In previous work, we developed live-cell, time-lapse microscopy methods for hypersaline-adapted archaea to overcome the challenges of rapid salt crystallization on microscopy slides (27). Salt-impregnated agarose microchambers were fabricated using soft lithography, which support up to six generations of growth for *Hbt. salinarum.* Using these tools, we demonstrated that single, rod-shaped *Hbt. salinarum* cells grow (elongate) exponentially, adding a constant volume between divisions (the “adder” model of cell size control [28]). However, the size distribution and division site placement at midcell demonstrated greater variance than bacterial cells that maintain their size in a similar fashion (27). Here, we adapt microfluidics for *Hbt. salinarum* and leverage the existing genetics and systems biology toolkits to interrogate the regulation of the archaeal cell cycle.

Cell cycle progression in eukaryotes is known to be exquisitely regulated, and DNA replication and cell division are coordinated in bacteria (29). However, despite recent progress regarding cell growth and size control in archaea, the underlying molecular mechanisms regulating these processes remain unknown. Gene expression profiling experiments suggest that archaea possess the capability for oscillating gene expression patterns, a hallmark of genes with cell cycle-related functions in eukaryotes (30). For example, our prior work with transcriptomics in *Hbt. salinarum* provides evidence for temporally coordinated induction of hundreds of genes during the resumption of growth following stasis (31). Oscillating gene expression was observed in *Hbt. salinarum* cultures entrained to day-night cycles (32). Cyclic gene expression patterns have also been observed in synchronized cultures of the crenarchaeon Sulfolobus solfataricus (3).

Gene regulatory networks (GRNs), comprised of interacting transcription factors (TFs) and their target genes, are central to the process of dynamic, physiological response to a variable environment. Archaeal transcription proteins resemble those of both bacteria and eukaryotes at the level of amino acid sequence. Basal transcriptional machinery required for transcription initiation in archaea, like that of eukaryotes, consists of transcription factor II B, a TATA binding protein, and an RNA-Pol II-like polymerase (reviewed in reference 33). The proteins that modulate transcription (e.g., activator and repressor TFs) typically resemble those of bacteria, with the majority of these proteins possessing helix-turn-helix (HTH) or winged-HTH DNA binding domains (34). Our recent studies on GRNs in *Hbt. salinarum* systematically investigated the function of transcription factors using high-throughput phenotyping of TF knockouts (35, 36). This study implicated the putative TF DNA binding protein VNG0194H (VNG_RS00795) as a candidate regulator of multiple stress responses: deletion of *VNG0194H* led to a growth defect under multiple stress conditions, including oxidative stress, low salinity, and heat shock (35). Intriguingly, the *VNG0194H* gene is located upstream of *ftsZ2* (37), suggesting additional roles for VNG0194H in cell growth and/or division. An additional putative DNA binding transcriptional regulator VNG0195H is encoded upstream.

To address knowledge gaps regarding archaeal cell division mechanisms, we investigated here the cell growth and division functions of FtsZ2, VNG0194H (CdrS [cell division regulator short]) and VNG0195H (CdrL [cell division regulator long]). We combine a battery of assays, including genetic knockouts, quantitative time lapse microscopy of single cells, custom microfluidics technology, gene expression profiling, and TF-DNA binding ChIP-seq experiments. The resultant data demonstrate that CdrS and FtsZ2 are required for normal cytokinesis but not cell elongation. This regulation is accomplished via (i) CdrS activation of *ftsZ2* and other cell cycle-related genes and (ii) CdrL direct regulation of the *cdrS-ftsZ2* operon. The CdrSL GRN system is highly specific to regulation of *ftsZ2* at the level of transcription.

## RESULTS

### *cdrS* encodes a conserved, putative transcription factor co-transcribed with the tubulin-encoding *ftsZ2* gene.

**FIG 1:**
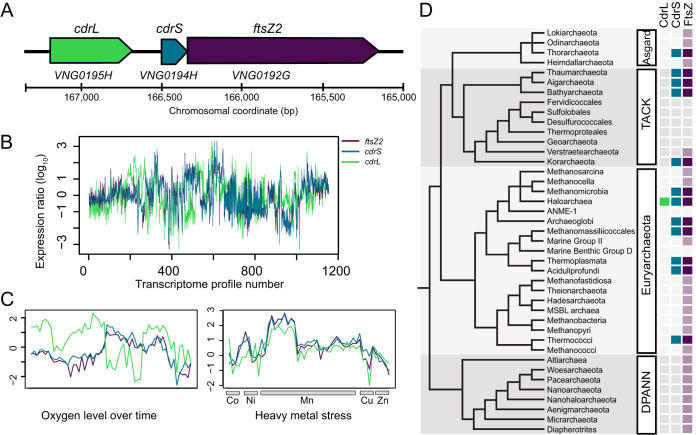
*cdrS* and *ftsZ2* comprise a polycistronic locus conserved throughout archaeal genomes. (A) Locus organization of *cdrL*, *cdrS*, and *ftsZ2* genes (*VNG_RS00800* to *VNG_RS00790*; *VNG0195H* to *VNG0192G*). Genes are drawn to scale, with chromosomal coordinates and a scale bar shown below the locus diagram. (B) Log_10_ gene expression data from 1,154 normalized microarray experiments over various conditions and genetic backgrounds. The *x* axis indicates the transcriptome profile number across multiple growth and stress conditions (25, 26). The *y* axis represents the normalized log_10_ gene expression ratio relative to the wild-type control under optimum growth conditions (mid-logarithmic phase, rich CM medium, 37°C, 225-rpm shaking). See the legend for line colors. (C) Gene expression under subsets of conditions in which *cdrL* is not coexpressed (left) or is coexpressed (right) with *cdrS* and *ftsZ2*. Axes and line colors are as in panel B. (D) Cooccurrence of *ftsZ2* and *cdrS* in the major archaeal lineages (78–80). Light purple boxes indicate clades with genomes found to encode at least one FtsZ protein but no CdrS (see also Table S1). Blue and dark purple boxes indicate clades with genomes that encode genes homologous to *cdrS* and *ftsZ2* in synteny (see also Fig. S1).

Our previous genetics experiments indicated an important role for putative DNA binding protein VNG0194H in stress response and growth physiology of *Hbt. salinarum* (35, 36). We first used bioinformatics to generate hypotheses regarding the physiological function of VNG0194H and its encoding gene locus (Fig. 1A)*. VNG0194H* is predicted to encode a small 55-amino-acid, single-domain protein that exhibits >99% structural homology to other ribbon-helix-helix (RHH) domain transcriptional regulators of the RHH_1 family (Pfam accession PF01402, E value of primary sequence homology 5.3 × 10^−5^; 99.6% confidence in structural homology to transcription factor NikR), suggesting that it may function as a DNA binding transcriptional regulator or in protein-protein interactions (38). Encoded immediately upstream, the VNG0195H protein is predicted to contain an N-terminal RHH_1 domain (95.9% confidence in structural homology to NikR) and a C-terminal double zinc ribbon domain (DZR, PF12773, E value 3.1 × 10^−7^). The presence of VNG0195H in this locus appears to be unique to halophiles (see Fig. S1 [all supplemental material can be accessed via the FigShare repository associated with this study at https://doi.org/10.6084/m9.figshare.12195081]). In addition, a tubulin FtsZ homolog is encoded immediately downstream of the *VNG0194H* gene (*ftsZ2*; *VNG0192G*; Fig. 1A). *Hbt. salinarum* FtsZ2 exhibits strong primary sequence identity to known tubulin components of the cell division ring (tubulin/FtsZ GTPase domain PF00091, E value 7.4 × 10^−77^; tubulin C-terminal domain PF03593, E value 6 × 10^−32^ [18]). Taking these bioinformatic analyses together, we renamed VNG0194H as “CdrS” for “cell division regulator Short” and VNG0195H as “CdrL” for “cell division regulator Long.”

Previous tiling microarray studies indicated cotranscription of *cdrS* and *ftsZ2* but were inconclusive regarding the inclusion of *cdrL* in this operon (39). To further investigate the transcriptional status of this locus, we examined the expression of the three genes from 1,154 microarray and three RNA-seq transcriptome profiles for *Hbt. salinarum* grown under a wide variety of environmental and genetic perturbations (25, 26) (Fig. 1B). Across all conditions, *cdrS* and *ftsZ2* were strongly and significantly correlated (Spearman’s ρ = 0.923, 95% confidence interval [CI] = 0.915 to 0.932), whereas *cdrL* was weakly but significantly correlated with *cdrS* (ρ = 0.275, 95% CI = 0.221 to 0.327) and *ftsZ2* (ρ = 0.299, 95% CI = 0.245 to 0.350). As a control, we calculated the correlation of these genes with an unrelated gene located elsewhere in the genome (*trmB VNG1451C*), which exhibited weakly negative correlation with the locus (*cdrS*, ρ = −0.275, 95% CI = −0.327 to −0.221; *ftsZ2*, ρ = −0.291, 95% CI = −0.343 to −0.238). The weak but significant correlation of *cdrL* with *ftsZ2* and *cdrS* is driven by strong coexpression of the three genes in response to stress such as metal overload (50 transcriptome profiles, ρ = 0.771, CI = 0.627 to 0.864; Fig. 1C, right). In contrast, *cdrL* is not coexpressed with *ftsZ2* or *cdrS* under conditions that foster rapid growth (58 profiles; ρ = −0.158, 95% CI = −0.400 to 0.104; Fig. 1C, left). Previous statistical models that inferred the global gene regulatory network of *Hbt. salinarum* also predicted coregulation of *cdrS* and *ftsZ2* under all growth conditions, whereas *cdrL* was only coregulated with the other two genes under a subset of conditions (25). Together, these results suggest that *cdrS* and *ftsZ2* are cotranscribed from a polycistronic operon that is coregulated under all growth conditions. *cdrL*, in contrast, is conditionally coregulated with the other two genes.

To determine how broadly this locus is conserved outside *Hbt. salinarum*, we investigated the sequence conservation of each gene and the genomic synteny of the gene pair across archaeal genomes. The cooccurrence of *cdrS* with *ftsZ2* homologs was detectable across representatives of all known archaeal clades except DPANN (Fig. 1D; see also Table S1 in the supplemental material). FtsZ in the absence of CdrS was also widely distributed. Conservation of the *cdrS-ftsZ2* locus was particularly strong across the Euryarchaeota, with wide conservation across the halophilic archaeal clade (including the classes Halobacteria, Natrialbales, and Haloferacales) and neighboring phylogenetic class Methanomicrobia (see Fig. S1). CdrL was widespread across the Halobacteria but absent from all other archaeal clades. Taken together, these results suggest that (i) *cdrS* exhibits a strong primary and secondary structural homology to transcriptional regulators of the RHH family; (ii) the *cdrS-ftsZ2* locus encodes a highly conserved, coregulated transcriptional unit; and (iii) CdrL is a putative transcription regulator unique to halophiles and appears to be conditionally coexpressed with *cdrS-ftsZ2*.

### CdrL is a specific and direct regulator of the *cdrS-ftsZ2* operon.

**FIG 2:**
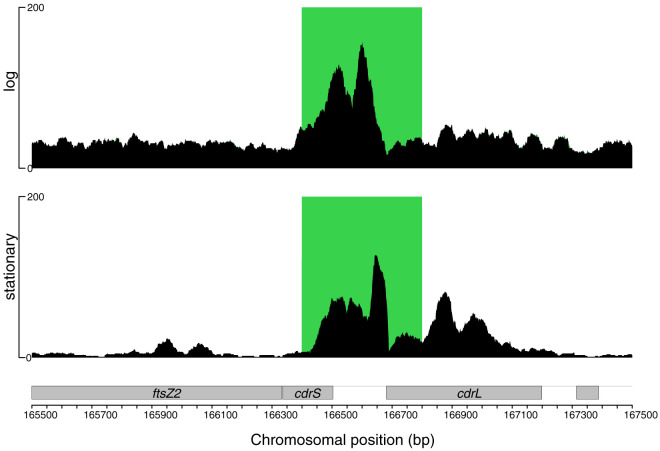
CdrL binds to the promoter region upstream of the *cdrS-ftsZ2* operon. Raw sequencing data for immunoprecipitated samples are indicated by the black traces. Overlaid green boxes represent genomic regions detected by the MOSAiCS peak detection algorithm (see Materials and Methods) to be significantly enriched for binding of CdrL relative to the input control. The *y*-axis scale represents read counts. The CdrL binding site is shown for logarithmic-phase cells (top) and stationary-phase cells (bottom). Gray labeled boxes at the bottom represent genes (reverse strand).

To further investigate how the *cdrS-ftsZ2* locus is regulated, we conducted protein-DNA binding analysis by chromatin immunoprecipitation coupled to sequencing (ChIP-seq; see Materials and Methods). Given the synteny and *cdrL* conditional coexpression with *cdrS-ftsZ2*, we reasoned that CdrL may play a role in regulation of the locus. The FLAG epitope was integrated into the chromosome at the 3′ end of the native *cdrL* locus, with the resultant strain encoding a C-terminal CdrL-FLAG translational fusion (see Materials and Methods; see Table S2). In both the mid-logarithmic and the stationary phases of growth, the region upstream of the *cdrS-ftsZ2* locus was the only significant CdrL binding site reproducibly detected throughout the genome (Fig. 2). Significant binding at other locations was detected in some ChIP-seq samples. However, these binding events were detected in genomic regions with redundant genes or poor coverage in the input sample. Other binding regions were not reproducible across replicate samples (see Fig. S2). We conclude that CdrL is a specific and direct regulator of *cdrS-ftsZ2* expression, binding exclusively and reproducibly upstream of this locus.

### The *cdrS-ftsZ2* locus is important for maintaining cell size and biomass in bulk culture.

To test the function of the *cdrL*-*cdrS-ftsZ2* locus, we constructed independent gene deletion mutants in each coding region. We previously reported a Δ*VNG0194H* strain (35, 36); however, that strain included a start site for the *ftsZ2* gene that was misannotated in the NCBI database, which we have corrected here (Fig. 1A; see also Fig. S1). This enabled a more precise, conservative deletion within *cdrS* to avoid polar effects by keeping the putative *ftsZ2* ribosome binding site intact (Fig. 1A; see also Tables S2 to S4). Because halophilic archaea are highly polyploid (40), stringent quality controls were implemented for both Δ*cdrS* and Δ*ftsZ2* strains, including PCR, Sanger sequencing, and whole-genome Illumina resequencing (see Materials and Methods). These controls confirmed that the *cdrS* and *ftsZ2* coding genes were removed from all genome copies and that no second-site mutations had accumulated (Table S5). However, second-site mutations were detected in the Δ*cdrL* mutant (Table S5). We therefore focused our remaining analysis on CdrS and FtsZ2.

**FIG 3:**
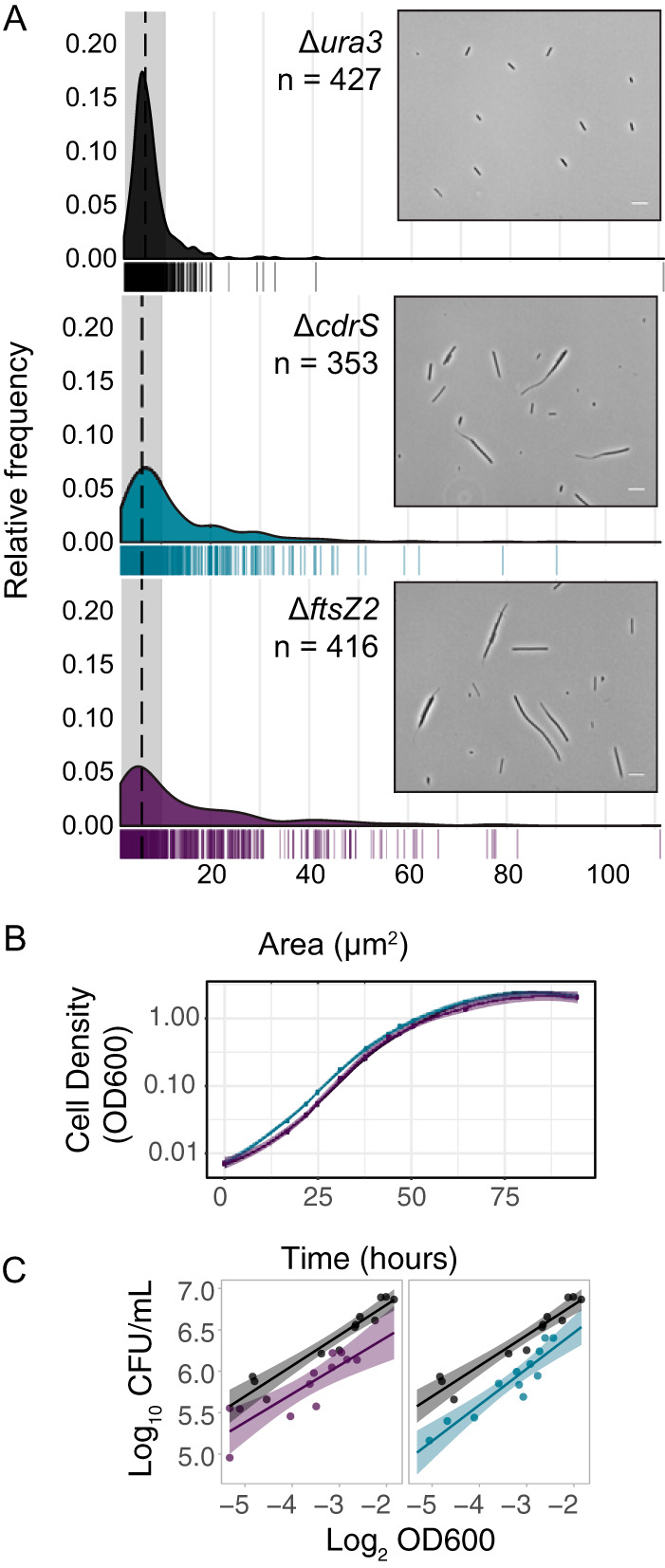
CdrS and FtsZ2 are required for maintaining cell size and division in batch culture but are dispensable for growth rate. (A) Area of individual cells during early exponential phase across Δ*ura3* (black), Δ*cdrS* (blue), and Δ*ftsZ2* (purple) strains. The median area for Δ*ura3* is indicated by a black dashed line. Gray shading indicates one standard deviation flanking the Δ*ura3* median in both directions. Insets show representative phase-contrast micrographs of the Δ*ura3* parent strain and mutants during early exponential growth. White scale bar, 10 μm. (B) Growth curve for all strains in rich media with aerobic conditions measured using the OD_600_. Solid lines represent the mean of three independent biological replicate samples, and shaded regions represent 95% confidence intervals. Black, Δ*ura3*; blue, Δ*cdrS*; purple, Δ*ftsZ2*. (C) Correlation of cell concentrations by CFU per ml and OD_600_. Dots in each panel represent quantification at multiple time points sampled from three replicate exponentially growing batch cultures for each strain. Solid lines indicate the linear regression fit to the data points, with shaded regions representing the 95% confidence intervals. The colors are consistent with panels A and B.

To investigate the phenotypes of the Δ*cdrS* and Δ*ftsZ2* mutant strains, cells were grown in aerobic batch culture in rich medium. Population growth rates, colony forming units (CFU), and single cell length and area were quantified (see Materials and Methods). Early-exponential-phase cultures of the Δ*ura3* parent strain were comprised of cells with a mean length of 5.39 μm (σ = 3.076 μm; Table 1; see also Table S6 in the supplemental material) and an area of 6.32 μm^2^ (σ = 4.12 μm^2^; Fig. 3A, top panel). A total of 87.9% of the Δ*ura3* cells fell within one standard deviation of the geometric mean length (Fig. 3A, insets; Table 1). These length and area measurements are consistent with previous observations for *Hbt. salinarum* growth and division (27). Also, 10.3% of the cells were longer than one standard deviation above the geometric mean; these longer cells in the rightward skew of the distribution are commonly seen during routine culturing and contribute to the noise in the *Hbt. salinarum* cell division model (27). Similar cell lengths were measured at time points sampled in mid-log phase and stationary phases of the growth curve (Table 1; see Fig. S3). In contrast, early exponential-phase cultures of the Δ*cdrS* strain cells were significantly longer (Welch’s *P* < 2 × 10^−16^; medium effect size, 0.760) and larger in area (*P* < 2.67 × 10^−16^; medium effect, 0.665) than those of the parent strain, with the longest cells being >40 μm. Large variation in size distribution of mutant length and area were also detected (Table 1; Fig. 3A, middle panel; see also Table S6). Similarly, the Δ*ftsZ2* strain cell size was significantly larger and more variable than that of the parent strain (length, *P* < 2.2 × 10^−16^; large effect, 0.832; area, *P* < 3.52 × 10^−15^, medium effect, 0.571; Fig. 3A, bottom panel). These differences in cell sizes between the parent and mutant strains were consistent across growth phases. Together, these microscopy results indicate a role for *ftsZ2* and *cdrS* in maintaining wild-type cell size in *Hbt. salinarum*.

**TABLE 1:** Cell area geometric means during batch culture (μm^2^ ± σ)

Parameter	Mutation	Geometric mean (μm^2^ ± SD) in various growth phases			
		Early exponential	Midexponential	Early stationary	Stationary
Length	Δ*ura3*	5.39 ± 3.08	5.72 ± 3.15	5.27 ± 2.76	5.09 ± 2.99
	Δ*cdrS*	8.67 ± 8.70	7.52 ± 8.02	5.23 ± 5.48	5.48 ± 4.15
	Δ*ftsZ2*	9.56 ± 10.04	7.65 ± 9.46	5.75 ± 6.52	5.24 ± 4.65
Area	*Δura3*	6.33 ± 4.12	6.73 ± 5.48	5.94 ± 4.42	6.21 ± 4.90
	*ΔcdrS*	9.50 ± 11.74	8.97 ± 16.31	6.16 ± 9.19	6.40 ± 7.53
	*ΔftsZ2*	9.76 ± 16.09	7.18 ± 14.12	6.14 ± 10.90	5.64 ± 8.64

However, it remained unclear whether the drastic increase in cell size together with morphology defects in Δ*ftsZ2* and Δ*cdrS* mutants were due to unregulated growth (cell elongation or biomass accumulation) or a decrease in cell division (fewer septation events). To compare the rate of biomass accumulation between wild-type and mutant strains, we measured growth rates by determining the optical density at 600 nm (OD_600_) for each of the parent, Δ*ftsZ2*, and Δ*cdrS* strains in batch culture under aerobic conditions in rich media (see Materials Methods). The maximum instantaneous growth rate of the Δ*ura3* strain was 0.152 ± 0.004 h^−1^ (see Table S6), consistent with previous observations (35, 36). Neither the Δ*cdrS* nor the Δ*ftsZ2* mutant strain exhibited a growth rate defect measured by OD_600_ and reached similar carrying capacities (OD_600_ = 1.73 to 2.62 for all three strains after 94 h; see Table S6; Fig. 3B). This suggests that deletion of neither *cdrS* nor *ftsZ2* reduces biomass as measured from the OD.

However, in the spectrophotometer, elongated cells scatter light differently than short cells (41), which can obfuscate true defects in cell size and/or division. Therefore, in addition to OD readings, we also plated for CFU. As the largest range of cell lengths occurred during early exponential phase, we plated multiple time points in the linear OD range between lag phase and an OD of 0.2. We detected a strong and significant positive correlation between log_2_-transformed OD and log_10_-transformed CFU/ml for the Δ*ura3* parent strain (Pearson’s ρ = 0.9561, *P* = 1.188 × 10^−6^; Fig. 3C). Similar correlations were detected for each of the Δ*cdrS* (ρ = 0.8166, *P* = 1.19 × 10^−3^) and Δ*ftsZ2* (ρ = 0.8509, *P* = 4.494 × 10^−4^) strains. These strong correlations enabled direct comparison between strains of the CFU normalized by the OD (see Materials and Methods). The Δ*ftsZ2* strain yielded 2.2-fold fewer CFU/ml per log_2_(OD) compared to the Δ*ura3* parent, and the Δ*cdrS* strain had 2.6-fold fewer. These results suggest that fewer viable individual cells are present in cultures of the mutant strains compared to the Δ*ura3* strain, which is likely the result of larger mutant cell size (more biomass per CFU). Together, these colony counts, cell density, and quantitative microscopy results support the hypothesis that *cdrS* and *ftsZ2* gene products are important for cell division but not cell area increase in batch culture.

### Δ*cdrS* cells are phenotypically insensitive to aphidicolin.

**FIG 4:**
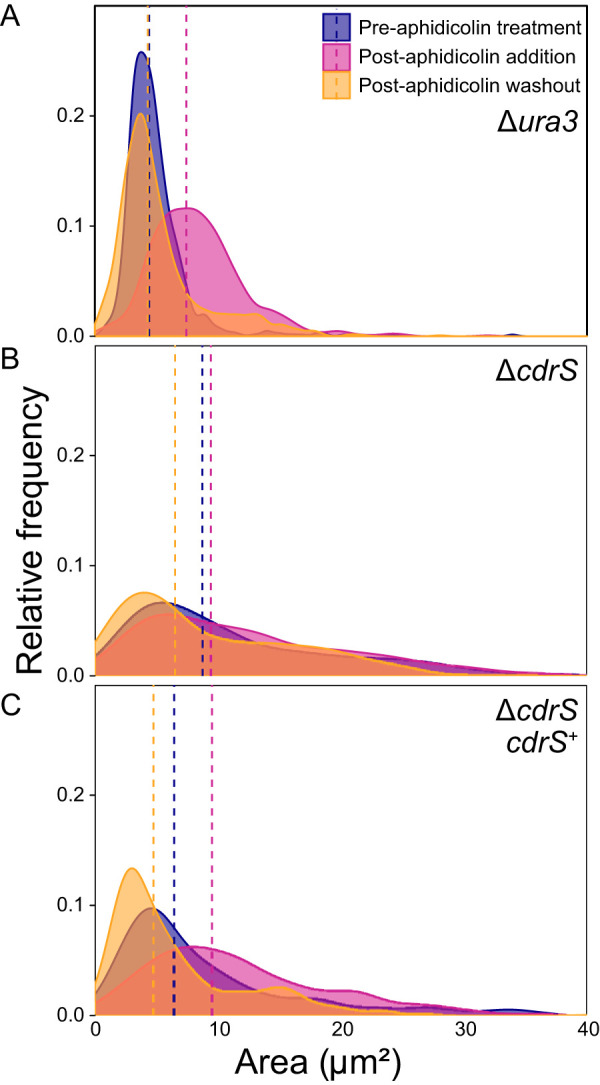
Δ*cdrS* is insensitive to cell cycle inhibitor aphidicolin. Cell area distributions are shown for the Δ*ura3* parent strain (A), the Δ*cdrS* strain (B), and the complementation strain (C). Colors (as indicated in the legend): blue, before drug addition; pink, after 6 h of exposure to aphidicolin; orange, 11 h after removal of the drug by washing. Dotted lines indicate the geometric mean for each time point.

To further test whether growth and cell division are decoupled in the Δ*cdrS* mutant, we synchronized populations of cells by treating with the cell cycle inhibitor aphidicolin, which specifically targets DNA polymerase α in eukaryotes (42). Aphidicolin has been shown to impair DNA replication and cell division but not elongation in wild-type *Hbt. salinarum* (15, 43, 44). Here, we quantified cell area prior to aphidicolin addition, after 6 h of cell cycle block in the presence of drug, and 11 h after drug removal (Fig. 4). After aphidicolin treatment, the Δ*ura3* average cell area increased significantly from 4.42 to 7.44 μm^2^ (geometric mean; Fig. 4A; *P* < 2.0 × 10^−16^; large effect size [ES] of 1.146), suggesting continued elongation in the absence of division and consistent with previous observations (15). After aphidicolin removal, Δ*ura3* cells returned to an average area indistinguishable from that of pretreatment values (4.31 μm^2^; *P* = 0.59; negligible ES 0.026). Cells undergoing septation were also observed, indicating a recovery of cell division (see Fig. S4). In contrast, the distribution of the Δ*cdrS* cell area remained largely unchanged by aphidicolin treatment (geometric mean of 8.73 μm^2^ before addition and 9.43 μm^2^ after 6 h of treatment, respectively; Fig. 4B; *P* = 0.15; negligible ES 0.094). Removal of aphidicolin from Δ*cdrS* cultures slightly decreased the cell area relative to the pretreatment area (6.52 μm^2^; 2.3 × 10^−7^; small ES 0.433); however, it is unclear whether this decrease is a biological or technical effect, since longer cells may shear during wash steps. Nevertheless, the distribution of cell areas in the Δ*cdrS* mutant remained heavily skewed toward elongated cells compared to the Δ*ura3* parent.

To ensure that the insensitivity to aphidicolin was specific to the deletion of *cdrS*, we generated a complementation strain by integration of P*_cdrS_-cdrS* into the chromosome at a neutral locus (NC_002607.1, 1245981 to 1247318; see Materials and Methods) (23). Prior to aphidicolin treatment, the complemented strain cell area was intermediate between that of the Δ*ura3* parent and Δ*cdrS* mutant strains (6.45 μm^2^), suggesting partial complementation (Fig. 4C, Table 2). However, after exposure to aphidicolin, the average cell area increased to 9.52 μm^2^ (*P* < 3.9 × 10^−14^, small ES 0.448, Table 2). After aphidicolin washout, the cell area mean returned to a smaller area of 4.78 μm^2^ (2 × 10^−16^, large ES 0.887). These shifts in distribution across the time course indicate that the complemented strain was responsive to aphidicolin, suggesting that complementation was achieved under native transcriptional control at the ectopic site. Therefore, while the area of the Δ*ura3* and the *cdrS* complementation strain were affected by aphidicolin treatment, the Δ*cdrS* mutant remained insensitive. Consistent with results from batch culture growth, these data suggest that CdrS is important for cell division but not elongation. In addition, given that aphidicolin treatment causes elongation of Δ*ura3*, phenocopying Δ*cdrS*, these data support the idea that *cdrS* may act as a checkpoint regulator, acting in the pathway that arrests cell division when DNA replication is perturbed.

**TABLE 2:** Geometric means of cell lengths during aphidicolin addition and removal

Strain	Mean cell length (μm) ± SD		
	Time zero	6 h after drug addition	11 h after drug removal
*Δura3*	4.42 ± 2.77	7.44 ± 3.81	4.31 ± 3.63
*ΔcdrS*	8.73 ± 9.05	9.43 ± 8.55	6.53 ± 7.07
*ΔcdrS ura3*::P*_cdrS_*-*cdrS*	6.45 ± 7.68	9.52 ± 7.42	4.77 ± 5.54

### Time-lapse microscopy reveals cell division defects in Δ*cdrS* and Δ*ftsZ2* mutants.

**FIG 5:**
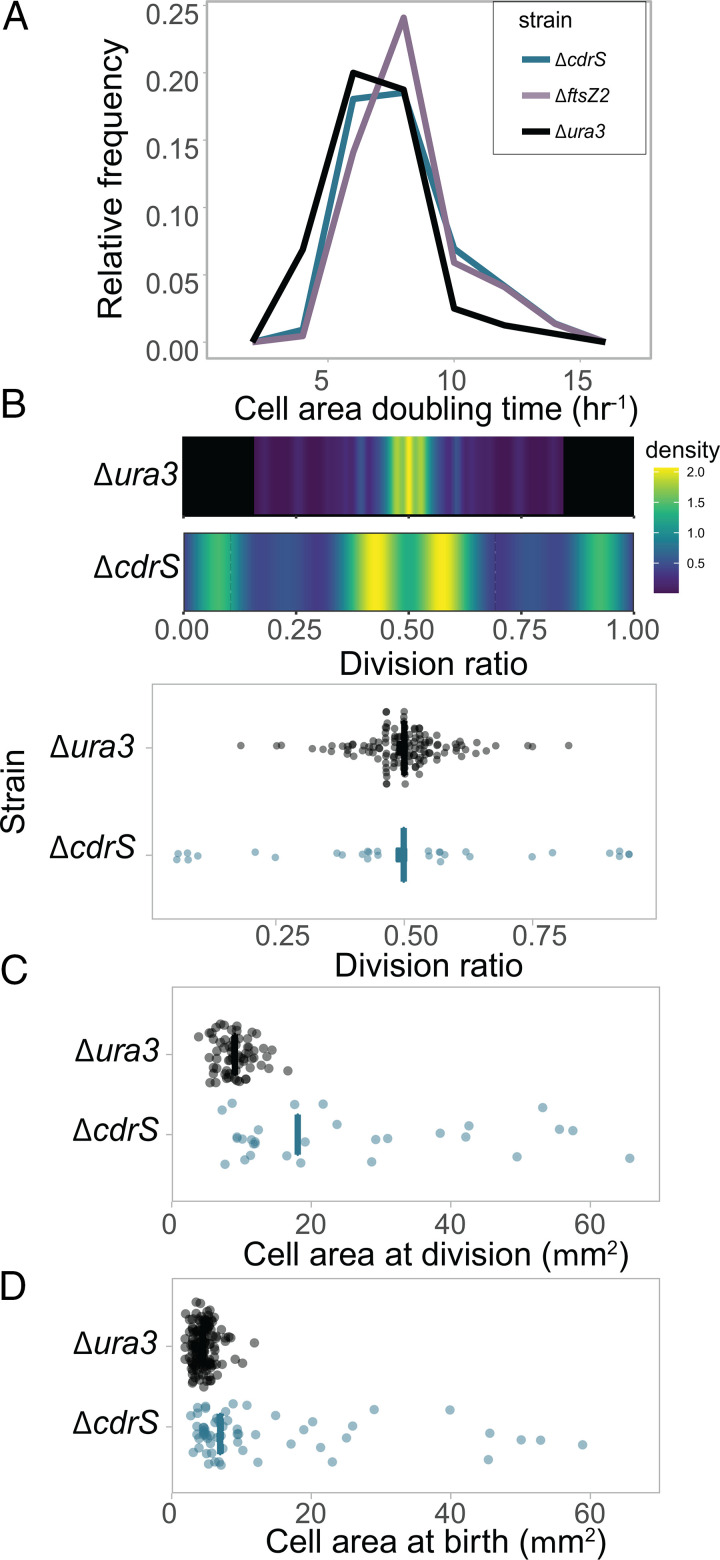
CdrS is required for division accuracy but not elongation. (A) Doubling time frequency plots for Δ*ura3* (black line), Δ*ftsZ2* (purple line), and Δ*cdrS* (green line). Legend colors are consistent throughout the figure. (B) Heatmap depicting the density distributions of division ratios for Δ*ura3* (top) and Δ*cdrS* (bottom) strains. Cool colors represent low density; hot colors represent high density (see the scale at the right). A raw data dot plot is shown below. The vertical crossbar represents the geometric mean. (C) Dot plot depicting area of mother cells at the time of division. The crossbar represents the median. (D) Dot plot depicting area of daughter cells immediately following division. A raw data dot plot is shown below. The crossbar represents the median.

In previous work, we designed agarose chambers that allowed real-time microscopic observation of unperturbed growth of *H. salinarum* (27). However, these chambers were square (10 by 10 μm), precluding growth of the filamentous Δ*ftsZ2* and Δ*cdrS* strains and necessitating a new device. We adapted the mother machine microfluidic device for real-time growth observation of these mutant *Hbt. salinarum* strains. The mother machine design used for hypersaline adapted archaea here is the same as those used previously for bacteria (45), consisting of linear channels 1.5 μm wide, 1 μm deep, and a selection of lengths (80 μm used here; see Fig. S5). The mold for the chip was fabricated via photolithography. The chip is arrayed with ∼200 troughs per feeding channel (four feeding channels per slide). Each chip was connected to microfluidics, supplying the growing cells with fresh medium throughout the course of each experiment (see Materials and Methods). Under steady-state growth conditions in the mother machine, the Δ*ura3* cell area doubling time (6.85 ± 1.98 h; Fig. 5A) was similar to that in batch culture (6.68 h; Fig. 1B; see Table S6). Mother machine growth rates also reflected previous chamber growth measurements (6 ± 1 h) (27). Like the square chambers, the mother machine supports up to 6 generations of growth (see Movie S1 in the supplemental material). We conclude that the *Hbt. salinarum* parent strain grows optimally in the mother machine.

To compare division events from single cells across strains in real time using the mother machine, phase-contrast time-lapse images were used to quantify the cell growth parameters of elongation rate, division ratio, and interdivision time. The cell area doubling times (proportional to elongation rate) of the Δ*cdrS* (geometric mean, 7.88 h ± 2.21 h) and Δ*ftsZ2* (8.04 ± 2.03 h) mutants were statistically indistinguishable from the Δ*ura3* parent strain (6.86 ± 1.98 h; Fig. 5A; see Fig. S6; *P* values comparing each mutant to the parent of >0.76; effect size, 0 to 0.1). This corroborates batch culture results that CdrS and FtsZ2 are not required for cell elongation. In contrast, division was strongly impaired for each mutant relative to the parent strain. Division of the Δ*cdrS* strain (30 of 108 cells divided) was observed at 35% the frequency of Δ*ura3* (63 of 80 cells), and the Δ*ftsZ2* strain was not observed to divide (0 of 110 cells). Δ*cdrS* cells that did not divide continued to elongate throughout the experiment, filling the chamber. In some cases, growth continued after the cell pole was extruded outside the single cell trough (see Movie S2 in the supplemental material). Typically, Δ*ura3* cells divided in the center, with the division ratio (area daughter to area mother) centered at 0.492 (coefficient of variance [CV] of 17.6%), which was quantitatively consistent with previous observations (27). In contrast, Δ*cdrS* cells divided asymmetrically, with few cells observed to divide in the center (Fig. 5B). This asymmetric division pattern was not random: 30% divided nearby the cell pole (division ratio, ≤0.20), 50% divided offset from the cell midline (i.e., division ratio, 0.3 to 0.6), a few outliers at the cell quarters (∼0.25 or 0.75), and none dividing within 0.05 μm of the center (Fig. 5B). The mean area of the Δ*cdrS* mother cells at the time of division (geometric mean, 19.64 μm^2^; σ = 17.57) was significantly larger and more variable than that of Δ*ura3* (Fig. 5C; 8.79 μm^2^; σ = 2.52; Welch’s *P* = 1.7 × 10^−7^; effect size, 1.50). Δ*cdrS* daughter cell mean area was twice as large as that of Δ*ura3* (Fig. 5D; 8.81 μm^2^; σ = 13.67 versus 4.32 μm^2^; σ = 1.60, respectively; Welch’s *P =* 2.3 × 10^−10^ and effect size 1.16), albeit the smallest Δ*cdrS* cells were within the same size range as Δ*ura3* cells (∼3 to 10 μm). This suggests that asymmetric division leads to variable cell sizes of mothers and daughters, with a tendency toward larger cell size in Δ*cdrS* relative to that of the Δ*ura3* parent (Fig. 5C and D).

**FIG 6:**
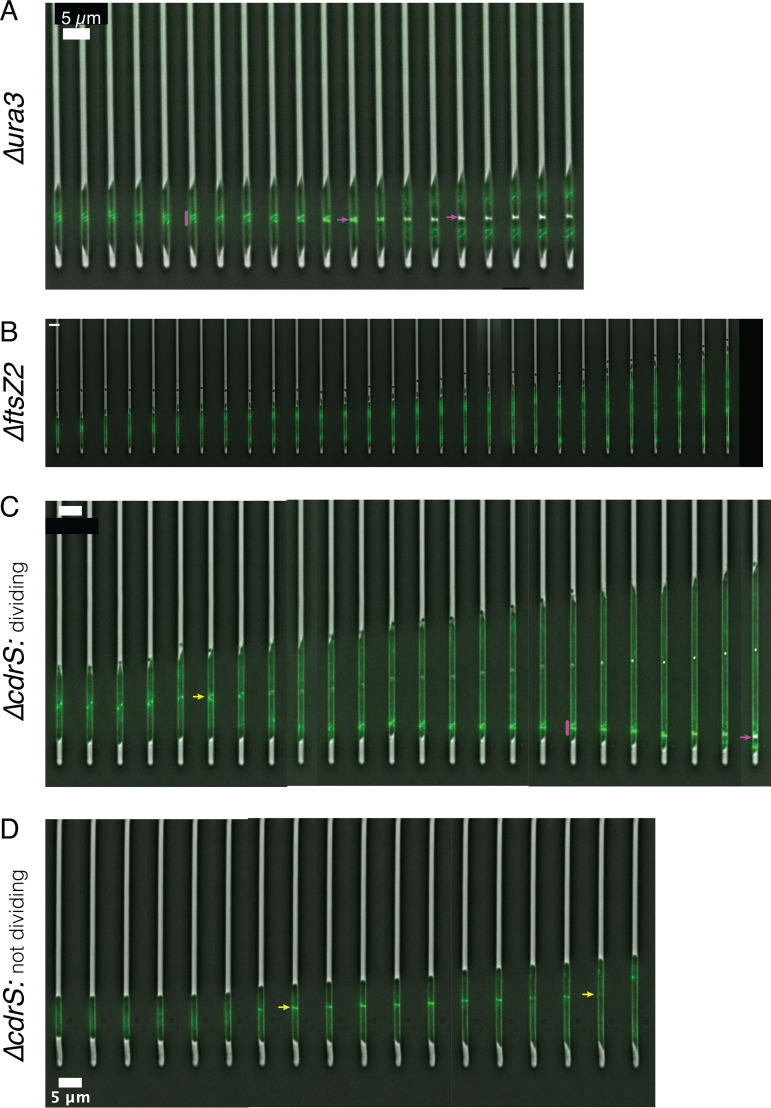
CdrS and FtsZ2 are important for triggering cytokinesis. (A) Montage of a representative Δ*ura3* cell growing in the mother machine. A pink vertical bar indicates the helical assembly of the division ring, and pink arrows indicate the division site just prior to and just after division. The montage corresponds with Movie S1 in the supplemental material. (B) Montage of a representative Δ*ftsZ2* cell growing and not dividing. No division ring is observed. The montage corresponds to Movie S3 in the supplemental material. (C) Montage of a representative Δ*cdrS* cell growing and undergoing polar division. The yellow arrow represents a division ring forming that later dissipates and does not result in division. The pink bar and arrow are as described in panel A. The montage corresponds to Movie S5a in the supplemental material. (D) Montage of a representative Δ*cdrS* cell that does not divide in the time frame of the movie. The yellow arrows represent a division ring forming and later dissipating, respectively. The montage corresponds to Movie SM5b in the supplemental material. For all panels, the time between each still image is 20 min, and the scale bars are 5 μm.

In the same cells visualized for quantitation with phase-contrast imaging, we tracked FtsZ1 division rings to differentiate active division events from cell fragmentation. The gene encoding monomeric superfolder GFP (msfGFP) was integrated at the native chromosomal locus by translational fusion to FtsZ1 in each of the three strain backgrounds (see Materials and Methods; see Table S2 in the supplemental material). In the Δ*ura3* strain, we observed that cell division was preceded by helical assembly of the FtsZ1 ring (Fig. 6A; see Movie S1 in the supplemental material). In contrast, deletion of *ftsZ2* abrogated ring formation in some cells, with a diffuse msfGFP-FtsZ1 signal observed throughout the cell (see Movie S3; Fig. 6B). In other Δ*ftsZ2* cells, division rings formed but constriction was not observed (see Movie S4). These rings appeared decondensed relative to the parent strain. Despite these division defects, Δ*ftsZ2* cells continued to elongate for the duration of the imaging experiments, filling the chamber (see Movie S3; Fig. 6B). For Δ*cdrS* cells that were able to divide (though at a lower frequency than Δ*ura3* cells), each division event was preceded by formation of a msfGFP-FtsZ1 ring (Fig. 6C; see Movie S5a in the supplemental material). However, not all ring formation resulted in division: in Δ*cdrS* cells that were not observed to divide, rings often formed but later disassembled (Fig. 6D; see Movie S5b in the supplemental material). Taking these fluorescence images together with the quantitative analyses of the phase-contrast images (Fig. 5), these data strongly suggest that CdrS is an important regulator of cell division, and FtsZ2 is required for triggering cytokinesis at midcell. However, elongation appears to proceed independently of these factors.

### CdrS specifically regulates cell division and other cell cycle genes.

**FIG 7:**
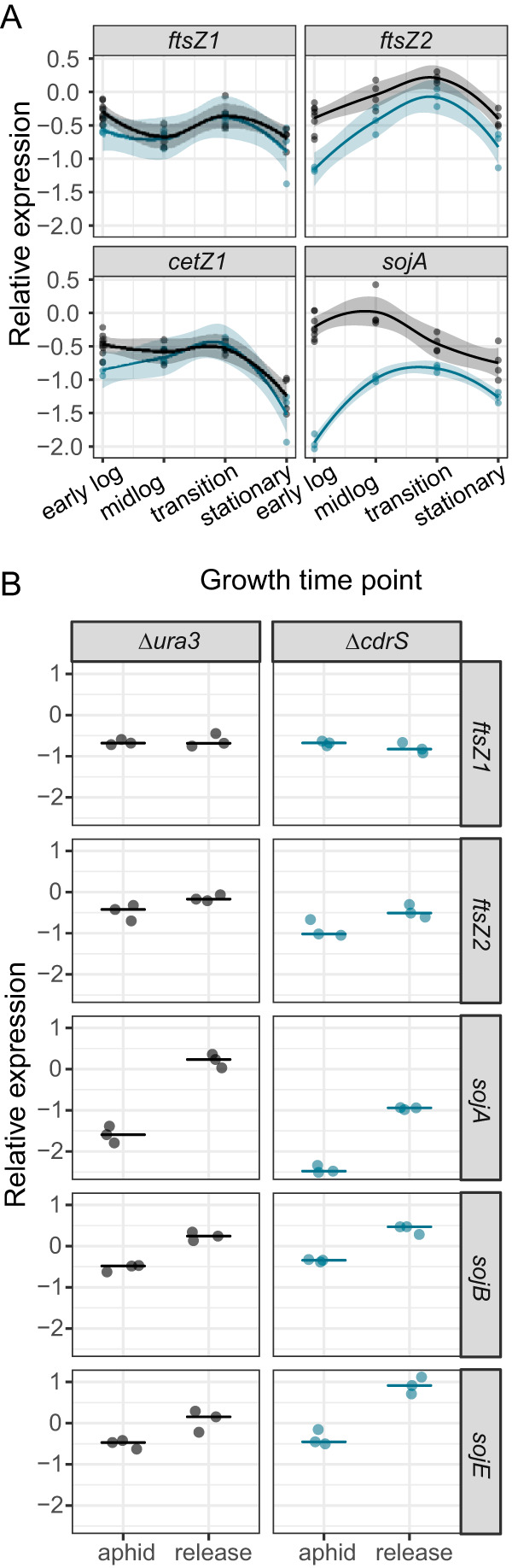
CdrS is important for wild-type expression levels of growth and cell division genes. (A) NanoString gene expression over the growth curve. Points represent log_10_-normalized expression data. Shaded regions represent smoothed conditional means. Labels on the *x* axis correspond to growth phases. (B) Expression data in response to cell division block with aphidicolin. Labels on *x* axis: aphid, gene expression during aphidicolin block; release, expression following washout. Points represent raw data; lines represent medians. Blue lines and points represent ∆*cdrS*; black, ∆*ura3* parent strain.

To determine how CdrS regulates cell division, we compared gene expression of the Δ*cdrS* strain to the isogenic parent Δ*ura3* over the growth curve and in response to cell cycle arrest by aphidicolin. Given the cell division defects of the Δ*cdrS* strain, we focused on 20 genes known or predicted to be involved in growth and division in other microbial systems, including all known *ftsZ* and *cetZ* paralogs encoded in the *Hbt. salinarum* genome. We used NanoString probe-based mRNA counting technology. Previous work demonstrated that this method is successful for accurate quantification of gene expression over time in *Hbt. salinarum*, enabling direct counting of mRNA molecules (46). Of the genes tested, 10 were significantly differentially expressed in the Δ*ura3* parent strain in batch cultures over the course of the growth curve, including *ftsZ2* and three CetZ homologs—*cetZ1* (*VNG1933G*), *cetZ2* (*VNG0265G*), and *cetZ5* (*VNG6260G*) (see Fig. S7A and Table S7 in the supplemental material). Relative to the Δ*ura3* control strain, *ftsZ2*, *cetZ1*, and *sojA* were significantly differentially expressed in response to *cdrS* deletion during growth (Fig. 7A). The protein product of plasmid-encoded *sojA* is a predicted member of the SIMIBI superfamily (NCBI accession cl28913), encompassing NTP-ases involved in a wide array of cellular functions, including the plasmid partitioning ParA AAA-type ATPase widely conserved in bacteria (47). *ftsZ1* was not differentially expressed either over the growth curve or in the Δ*cdrS* versus the parent strain (Fig. 7A, upper left). In contrast, *ftsZ2*, *cetZ1*, and *sojA* were dynamically expressed throughout growth, and expression levels were lower in Δ*cdrS* during early log phase (Fig. 7A). For example, *ftsZ2* expression steadily increased ∼1.8-fold throughout growth, reaching its peak during the transition to stationary phase (Fig. 7A, upper right; see also Table S7). In the Δ*cdrS* strain, *ftsZ2* expression followed a similar growth-dependent expression pattern. However, expression magnitude ranged from 1.3- to 2.2-fold lower across all growth time points in the Δ*cdrS* strain, with the largest defect in gene activation observed at the early log time point, with *cetZ1* and *sojA* following similar patterns (Fig. 7A).

In response to cell division block by aphidicolin and subsequent release into growth, 11 genes were significantly differentially expressed in the Δ*ura3* parent strain (see Fig. S7B and Table S7). *ftsZ2* expression was also significantly reduced in the Δ*cdrS* strain relative to the Δ*ura3* strain in response to aphidicolin (Fig. 7B). Three *par* family paralogs (*sojA*, *sojB*, and *sojE*) were also significantly misregulated in the Δ*cdrS* strain relative to the parent control under these conditions (Fig. 7B). Across both the growth and the aphidicolin treatments, we observed that absolute expression levels of *ftsZ2* were extremely high (mean count, >2 × 10^6^, Table S7), ranking as the third highest expression of the genes tested in the parent strain. In contrast, *ftsZ1* counts were 2 orders of magnitude lower (∼9,000, twelfth highest expression). Since *ftsZ2* but not *ftsZ1* expression was reduced in the Δ*cdrS* mutant, CdrS may act to maintain the normal balance between *ftsZ1* and *ftsZ2* levels. Together, these expression data indicate that CdrS is important for wild-type expression magnitude but not growth-dependent expression change of the *ftsZ2*, *cetZ1*, and SIMIBI family protein-coding genes. These results are consistent with the hypothesis that CdrS is a specific regulator of the cell division ring and other putative cell division-related functions.

### *cdrS* homologs in other *Haloarchaea* are required for maintaining cell shape and size.

**FIG 8:**
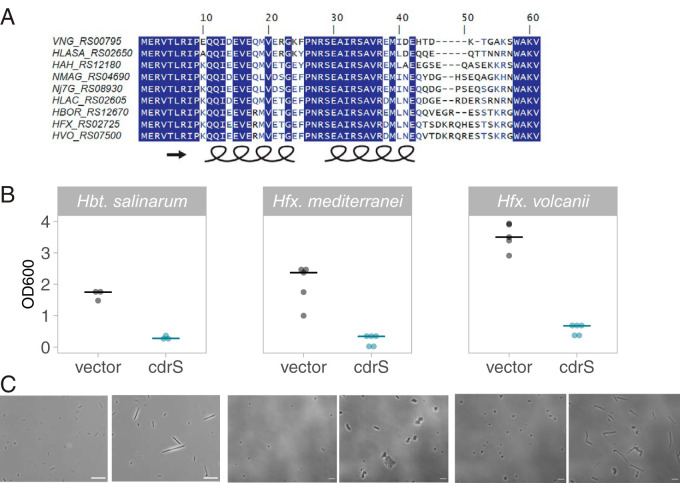
CdrS is required for cell division across halophilic archaea. (A) Clustal Omega alignment of protein sequences from halophilic archaeal model organisms. The GenBank protein sequence identifiers for CdrS homologs are given at left. Species identifiers are as follows: VNG, *Hbt. salinarum*; HLASA, Halanaeroarchaeum sulfurireducens; HAH, Haloarcula hispanica; NMAG, Natrialba magadii; Nj7G, *Natrinema* sp. strain J7-2; HLAC, Halorubrum lacusprofundi; HBOR, Halogeometricum borinquense; HFX, *Hfx. mediterranei*; HVO, *Hfx. volcanii*. (B) Final cell density measurements of overnight cultures of empty vector control strains (black) versus *cdrS*^+^ overexpression strains (blue) in *Hbt. salinarum* (left, *VNG0194H*, pAS0194_Chis), *Hfx. mediterranei* (middle, *HFX_0561*, pAKS77), and *Hfx. volcanii* (right, *HVO_0582*, pAKS78). (C) Phase-contrast micrographs comparing empty vector control to *cdrS*^+^ overexpression strains. Species and strain labels align in columns according to the labels in panel B.

The *cdrS-ftsZ2* locus was detected in all known haloarchaeal genomes (Fig. 1), and protein alignments showed strong conservation in model species across the clade (Fig. 8A). The beta-sheet region of the RHH protein was perfectly conserved, and only nine residues of the alpha-helical regions varied across these species (Fig. 8A). Given this strong conservation, we hypothesized that CdrS plays a conserved functional role as a regulator of cell division across hypersaline-adapted archaeal species. However, multiple attempts to delete *cdrS* in the genetically amenable model species Hfx. volcanii (HVO_0582; HVO_RS07500) and Hfx. mediterranei (HFX_0561, HFX_RS02725) were unsuccessful despite using a selection-counterselection scheme routinely used in the field (24, 48). In *Hfx. volcanii*, 48 clones were screened by PCR across three transformations, and 292 clones were screened by PCR across four transformations in *Hfx. mediterranei*. No *Hfx. mediterranei* knockout candidate clones were detected. Eight *Hfx. volcanii* candidates were identified; however, Sanger sequencing detected many point mutations throughout the locus. These results, corroborated by a parallel study on *cdrS* in *Hfx. volcanii* (V. Vogel et al., unpublished data), suggest that *cdrS* is required for viability under laboratory conditions in these species.

Instead, we overexpressed *cdrS* in these species to investigate how its role in cell division is conserved. We compared phenotypes to a *cdrS* overexpression strain of *Hbt. salinarum. cdrS* was cloned downstream of strong constitutive promoters in autonomously replicating vectors and transformed into the respective haloarchaeal species (49) (Tables S2 to S4). Overnight cultures of the empty vector control strain grew well under selection in both Haloferax species, reaching high final cell densities (OD_600_ of 2.0 to 3.5; Fig. 8B). Similarly, the Hbt. salinarum control strain reached mean final cell densities of 1.67 OD_600_ after 72 h of growth, as expected from previous research (50). In contrast, strains carrying the *cdrS* overexpression plasmid (*cdrS^+^*) exhibited significant growth inhibition in all three species, indicating the importance of tight control of *cdrS* expression levels (Fig. 8B). Like the *Hbt. salinarum* deletion strain, the overexpression strain is elongated and filamentous relative to the rod-shaped control (Fig. 8C). Similarly, in both *Haloferax* species, severe morphological defects were observed in *cdrS* overexpression strains compared to the disc-shaped control strain (Fig. 8C; see Fig. S8 in the supplemental material). *Hfx. volcanii cdrS^+^* overexpression cell area was, on average, 3-fold larger than the corresponding empty vector control strain (Welch’s *P* < 2.2 × 10^−16^; effect size, 1.355 [large]; see Fig. S8; Table 3). *cdrS*^+^ cells were also 3-fold longer than the disc-shaped control, suggesting that the area increase was primarily due to elongation of the cell body. However, thickness varied along the length of the *cdrS^+^* cells, often resulting in club-like shapes (Fig. 8C). Similar to *Hbt. salinarum* Δ*cdrS* (Fig. 3 and 5), the *cdrS^+^* cell area was more variable than that of the empty vector cells, suggesting impaired regulation (Table 3; see Fig. S8). Similar results were obtained with the *Hfx. mediterranei* system, with a 3-fold larger cell area and increased variance observed relative to the control strain (Welch’s *P* < 2.2 × 10^−16^; effect size, 1.536 [large]; Table 3; Fig. 8C; see Fig. S8). However, unlike *Hfx. volcanii cdrS^+^* overexpression cells, the *Hfx. mediterranei cdrS^+^* cells were, on average, only 1.5-fold longer than control cells. We observed that *cdrS*^+^ in *Hfx. mediterranei* exhibited two major forms: one with an increase in cell area across two planes, generating plate-like cells, and the other elongated and/or club-shaped (Fig. 8C). Taken together, these data suggest that CdrS is required for maintaining wild-type disc-like cell shape and size in other halophilic archaea. Given the similarity of these phenotypes with those of *Hbt. salinarum*, these data are consistent with the hypothesis that CdrS in both *Haloferax* species is also required for cell division regulation but may play an additional role in maintaining cell shape and viability.

**TABLE 3:** Summary statistics of cell area and length in *Haloferax* species

Parameter and species	Strain	Geometric mean	Median	σ	Number of cells
Area					
*Hfx. volcanii*	*cdrS^+^*	15.72	17.78	49.54	1,105
	Vector	5.90	5.92	2.96	2,554
*Hfx. mediterranei*	*cdrS^+^*	11.65	11.63	17.06	1,253
	Vector	4.30	4.38	1.60	2,291
					
Length					
*Hfx. volcanii*	*cdrS^+^*	9.03	10.20	8.20	1,105
	Vector	3.25	3.17	1.31	2,554
*Hfx. mediterranei*	*cdrS*^+^	5.33	5.28	3.55	1,253
	Vector	2.62	2.62	0.55	2,291

## DISCUSSION

Growth and division are precisely controlled to ensure the coordination of cellular events. However, such regulation thus far has been unexplored in archaea. In the present study, we identify and characterize the highly conserved CdrSL gene regulatory network (GRN). Quantitative microscopy on cells from bulk culture and single-cell time-lapse images demonstrates the requirement of CdrS for cell division in the model archaeal species *Hbt. salinarum* (Fig. 3 and 6). Specifically, deletion of *cdrS* or *ftsZ2* impairs cell division but does not affect cell elongation rate, providing strong evidence that the CdrS regulatory system and FtsZ2 itself are required for coupling cell growth and division. Intriguingly, while FtsZ2 is absolutely required for cell division, a small subset of Δ*cdrS* cells are still able to divide (Fig. 5 and 6), hinting that other mechanisms regulating archaeal cell division await discovery.

Our genetic evidence, transcript profiling, and protein-DNA binding suggest that regulation is achieved via CdrS transcriptional activation of genes that encode proteins predicted to function in critical aspects of cell division (Fig. 2 and 7). These include the cell division ring (FtsZ2), cell shape maintenance (CetZ1) (18), and DNA partitioning (SojABE). One caveat is that the *soj* genes are encoded on the *Hbt. salinarum* pNRC100 and pNRC200 megaplasmids. These genomic elements are subject to frequent copy number variation (26), so further evidence is needed to determine the definitive mechanism by which CdrS affects their expression. Nevertheless, CdrS exerts its effect on these genes in early log phase and after release from a chemical cell division block, suggesting that CdrS acts during the transition from stasis to growth. CdrL provides a second level of regulation by binding the region upstream of the *cdrS-ftsZ2* operon (Fig. 2). Together, these data suggest a mechanism by which the CdrSL system controls cell division.

Here, we show that FtsZ proteins have distinct but interrelated roles in cell division, which are reflected in their differential regulation by CdrS (Fig. 5 and 7). Previous work in halophilic archaea suggests that a large class of tubulin-like proteins, FtsZ and CetZ, function in cell division and cell morphology, respectively (13, 18). Here, we build on this knowledge, demonstrating that *ftsZ1* expression levels are independent of CdrS regulation and remain relatively constant at different growth rates (Fig. 7). FtsZ1 rings form just prior to cell division events (Fig. 6). In contrast, *ftsZ2* transcript levels fluctuate depending on the presence of CdrS, growth phase, and chemical perturbations, indicating a growth-sensitive mechanism of transcriptional regulation (Fig. 7). Although *ftsZ2* can be deleted in *Hbt. salinarum* (Fig. 3 and 6; see also Table S5), it is essential for triggering the constriction of the cytokinetic ring during exponential growth, further suggesting condition-specific functions for FtsZ2 (Fig. 6). Therefore, the multiple copies of FtsZ within the Halobacteria, and likely other Euryarchaeota, do not appear to be redundant but instead may represent a case of subfunctionalization. CdrS plays a key role in regulating the interrelated but separate functions of the two FtsZ proteins, as Δ*cdrS* cells fail to activate *ftsZ2* during rapid growth (early log phase), delaying cell division (Fig. 5 and 7). Given the independence of cell elongation from regulation by CdrS and FtsZ2 (Fig. 3 and 5) and that haloarchaeal cells grow by inserting new surface layer (S-layer) material at midcell (17), cell elongation and cytokinesis may occur at the same cell region (Z ring) but may be temporally sequential and separately regulated. CdrS appears to play a key role in coordinating these events.

Our results evoke a function for archaeal FtsZ proteins analogous with those required for chloroplast division in land plants and some bacterial species. In chloroplasts, FtsZ1 and FtsZ2 have interrelated but nonredundant functions in cell division. The two FtsZ homologs form copolymers, with one thought to be involved in divisome structure, the other involved in dynamic GTP turnover and constriction (51). Similarly, in the alphaproteobacterium Agrobacterium tumefaciens, although two FtsZ proteins copolymerize at midcell, only one is required for constriction (52). Recent results in *Hfx. volcanii* suggest colocalization of FtsZ1 and FtsZ2 at midcell (53). Consistent with these multi-FtsZ models of cell division, here we observed defects in *Hbt. salinarum* FtsZ1 ring assembly in the absence of FtsZ2 or CdrS, suggesting that FtsZ1 and FtsZ2 could also form copolymers whose stoichiometry is balanced by CdrS regulation.

The CdrS-FtsZ2 system is widely conserved across the Archaea at both the protein structural and functional levels, with CdrL restricted to the Halobacteria (Fig. 1 and 8). At the level of primary structure, the ribbon-helix-helix (RHH) CdrS protein is detected in every major known taxonomic group of archaea except DPANN (Fig. 1). Further, RHH protein CdrL is encoded in all sequenced members of Halobacteria, though often only annotated by the C-terminal double-zinc-ribbon (DZR) domain. The CdrS and CdrL clades are phylogenetically distinct within the RHH superfamily (PF01402, Fig. 1; see also Fig. S1), suggesting independent evolutionary history. Restriction of *cdrL* to the Halobacteria further supports this hypothesis. Therefore, we surmise that the locus acquired *cdrL* after the divergence of methanomicrobial and halobacterial ancestors.

CdrS is also conserved at the functional level, since we demonstrate that CdrS is required for proper cell division across multiple species of haloarchaea, including *Hbt. salinarum*, *Hfx. volcanii*, and *Hfx. mediterranei* (Fig. 8). This was corroborated independently in a recent study that also demonstrated that *cdrS* is an essential gene whose product is required for cell division in *Hfx. volcanii* (Vogel, unpublished data). Given the essentiality of *cdrS* in both *Haloferax* species and the polyploidy of Halobacteria, we have included whole-genome sequencing (WGS) as an essential step of strain construction (see Table S5 in the supplemental material). Particularly with seemingly essential genes, we have found WGS more sensitive than standard PCR and Sanger sequencing in detecting all copies of a target gene, as well as ruling out second-site mutations (see also reference 54). Taking our phylogenetic and genomics evidence together, we conclude that the CdrS-FtsZ2 system is widely conserved and important for cell division across hypersaline-adapted archaea.

## MATERIALS AND METHODS

### Bioinformatic prediction and phylogenetic analysis.

Protein structural predictions of CdrS and CdrL were conducted using the Phyre2 server (55) using default parameters, access date 2/12/2020. The top hit reported in the text was the structure RHH DNA binding domain of the NikR transcription factor (protein data bank [PDB] identifier 2BJ7; rscb.org, original structure published in reference 56). Protein primary sequence predictions are reported for protein families (PFAM; pfam.xfam.org) (57), E values of significance of the matches for each protein were found in the *Hbt. salinarum* genome database (37; https://baliga.systemsbiology.net/projects/halobacterium-species-nrc-1-genome/). Gene expression correlations in Fig. 1C were calculated and visualized using the corrplot and psychometric packages in the RStudio coding environment, R version 3.6.1. Synteny of the *cdrL-cdrS-ftsZ2* locus (37; identifiers *VNG0195H-VNG0194H-VNG0192G*; NCBI identifiers *VNG_RS00800-VNG_RS00795-VNG_RS00790*) was determined using the SyntTax database using default parameters (https://archaea.i2bc.paris-saclay.fr/SyntTax/) (58). All 384 archaeal genomes housed in the SyntTax database were searched with the FtsZ2 (VNG0192G) protein sequence of *Hbt. salinarum*. Detection of locus homologs and synteny for those genomes not included in SyntTax (*Bathyarchaeota*, *Korarchaeota*, Asgard, 20 genomes) were found using NCBI genomes database using BLAST to detect FtsZ2 homologs (sequence similarity cutoff >200 bits). Subsequent manual inspection in the NCBI genome browser (https://www.ncbi.nlm.nih.gov/genome/) detected synteny of the locus. Locus identifiers for *cdrS-ftsZ2* across 93 archaeal genomes and UniProt protein identifiers for FtsZ-family homologs (including FtsZ and CetZ-like proteins) in the absence of *cdrS* across 1,497 genomes are given in Table S1 in the supplemental material. The CdrS protein sequence alignments shown in Fig. 8 were determined using Clustal Omega with default parameters in the DNAstar MegAlign software package.

### Strains, plasmids, and primers.

Halobacterium salinarum NRC-1 (ATCC 700922) was the wild-type strain used in this study. Gene deletions and chromosomal integrations were performed using two-stage selection and counterselection homologous recombination in the Δ*pyrF* (Δ*ura3*) strain isogenic parent background, as described previously (23), updated in (59), and subject to whole-genome resequencing here (see Table S5, Sequence Read Archive PRJNA614648). Plasmids were constructed using isothermal assembly (60) and propagated in Escherichia coli NEB5α (see the primer list in Table S3 for details). Primers were ordered from Integrated DNA Technologies (Coralville, IA). Final strain genotypes were verified using site-specific PCR and Sanger sequencing by Eton Biosciences, Inc. (San Diego, CA), and genomic DNA extraction, followed by Illumina sequencing (see below). The plasmids used in cloning are presented in Table S4, and the resultant strains are presented in Table S2.

For overexpression studies, Haloferax volcanii DS2 and Haloferax mediterranei ATCC 33500 were the wild-type strains. Plasmids were constructed from pJAM809 using restriction enzymes NdeI and KpnI to remove the resident open reading frame (ORF) and replace it with the *cdrS* gene from each species. Δ*pyrE2* derivatives of *Haloferax* species (22, 24) were transformed with NEB5α-propagated plasmid. Due to concerns about the higher mutation rate in methylase-deficient E. coli, we opted not to passage plasmids through a Δ*dam* Δ*dcm* strain, in contrast to what is commonly done (61).

### Media and growth conditions.

*Hbt. salinarum* strains were routinely grown using CM medium (250 g/liter NaCl [Fisher Scientific]; 20 g/liter MgSO_4_·7H_2_O [Fisher Scientific]; 3 g/liter trisodium citrate [Fisher Scientific]; 2 g/liter KCl [Fisher Scientific]; 10 g/liter bacteriological peptone [Oxoid]; pH 6.8). Media were supplemented with 50 μg/ml uracil (Sigma) to complement the uracil auxotropy of the Δ*ura3* background. During knockout and integrant strain construction, the first stage of selection was performed on mevinolin (10 μg/ml; AG Scientific) plates (CM with 20 g/liter agar; Difco), and the second stage of counterselection on 5-fluoroorotic acid (300 μg/ml; ChemImpex) was performed in agar plates. All growth was performed at 42°C, and liquid cultures were shaken at 225 rpm under ambient light. Self-replicating *Hbt. sailnarum* plasmids were maintained using 1 μg/ml mevinolin in liquid culture. Self-replicating *Hfx. volcanii* and *Hfx. mediterranei* plasmids were maintained using 0.1 μg/ml novobiocin. E. coli was grown in LB medium with carbenicillin (50 μg/ml; Sigma) to maintain plasmids. Maximum instantaneous growth rates were calculated as described previously (50). Raw data are provided in Table S6 in the supplemental material.

For statistical analysis of the OD versus CFU data shown in Fig. 3, we noted that the slopes of the regression lines were similar between Δ*ura3* and each mutant, so we fit a linear model using the log_2_(OD) and genotype to predict the log_10_(CFU). In a two-way analysis of variance (ANOVA) test, we found no evidence of interaction between strain and OD versus CFU slope (Δ*ura3* versus Δ*cdrS*, *P* = 0.225; Δ*ura3* versus Δ*ftsZ2*, *P* = 0.95). Therefore, a new model was fit that constrained equal slopes for the regression line for each strain, allowing us to determine the difference in CFU/ml per unit log_2_(OD) between strains. These differences are reported in the text.

### gDNA extraction and Illumina sequencing.

*Hbt. salinarum* strains were grown to mid-logarithmic phase (OD_600_ ∼0.7), and 1 ml was pelleted by centrifugation. Pellets were stored at –20°C until processed. DNA was extracted using a phenol-chloroform method. Briefly, pellets were lysed in dH_2_O and treated with RNase A and proteinase K. DNA was extracted using phenol-chloroform-isoamyl alcohol (25:24:1; Fisher Scientific) in Phase Lock Gel tubes (QuantaBio) and ethanol precipitated. DNA pellet was resuspended in modified TE buffer (10 mM Tris-HCl [pH 8.0], 0.1 mM EDTA). Purified DNA was quantified using a NanoDrop system (Thermo Scientific) and sonicated in a Diagenode sonicating water bath for 20 cycles on high. DNA quality was assessed by using a bioanalyzer using a high-sensitivity DNA chip (Agilent). Samples were submitted to Duke Center for Genomic and Computational Biology core sequencing facility for adapter ligation with TruSeq (Illumina) adapters and library amplification. Samples were pooled and run in a single lane on an Illumina HiSeq 4000. 50-bp reads were assessed for quality using FastQC, and adapter sequences were trimmed using Trim Galore and Cutadapt. Reads were aligned to the *Hbt. salinarum* NRC-1 genome using Bowtie2 within the breseq package, as described previously (54, 62). The breseq results were analyzed for incomplete gene conversion, SNPs, and possible genomic rearrangements. The results are given in Table S5; the code is freely available on github (https://github.com/amyschmid/aglB-WGS-growth), and raw data are accessible through the sequence read archive (SRA) under accession number PRJNA614648.

### Microscopy and quantitative analysis of cell morphology.

***Cell culture preparation for microscopic imaging. (i) Deletion mutants.*** Strains Δ*cdrS*, Δ*ftsZ2*, and Δ*ura3* cells were collected at various stages throughout the growth curve (see Table S6 for the OD_600_ values at harvest).

***(ii) Aphidicolin treatment.*** Cells were cultured to stationary phase and then subcultured in 20 ml of CM medium supplemented with uracil to an OD_600_ of ∼0.01. Subcultures were incubated at 42°C with 225 rpm shaking until reaching an OD_600_ of ∼0.3, at which point 30 μM aphidicolin (30 mM stock in DMSO; Sigma) was added. After 6 h of incubation with drug, the remaining culture was washed twice by centrifugation (4,500 × *g*, 6 min), resuspension in fresh CM, and incubation for 20 min at 42°C with 225 rpm shaking. Samples were harvested for microscopy (5 μl) prior to aphidicolin addition, prior to washing, and 11 h after washing (release) from aphidicolin treatment. The rationale for the time point selection was based on a previously published study (15).

***(iii) Overexpression strains.*** Five biological replicate overnight cultures of *Hfx. volcanii* strain H26 harboring the pAKS78 plasmid and *Hfx. mediterranei* WR510 harboring the pAKS77 plasmid were grown in rich Hv-YPC medium supplemented with novobiocin to maintain the plasmid and 50 μg/ml uracil to complement the auxotrophy of the Δ*pyrE2* background. Image quantitation and significance of the difference between *cdrS*^+^ overexpression strain cells and empty vector control strain in *Haloferax* species was calculated as described above for knockout mutants and aphidicolin experiments. To obtain micrographs of the *Hbt. salinarum cdrS*^+^ overexpression Δ*ura3* strain harboring the pAKS0194-Chis plasmid, cultures were grown in rich CM medium supplemented with mevinolin to maintain the plasmid and 50 μg/ml uracil to complement the uracil auxotrophy.

***Microscopy.*** For microscopy, 5-μl portions of three to five biological replicate culture samples (grown as described above) were mounted on 1% agarose pads equilibrated with basal salt buffer (medium without peptone or yeast extract) overnight at room temperature, except that the *Hbt. salinarum cdrS*^+^ overexpression strain was imaged on wet-mount slides. Phase-contrast images were taken with a Zeiss Axio Scope.A1 microscope (Carl Zeiss, Oberkochen, Germany) and a PixeLINK CCD camera (PixeLINK, Ottawa, Canada) at ×40 magnification.

***Image quantitation and statistical analysis.*** Quantification of cell length and area was calculated using the Fiji distribution (63) of ImageJ (64) and plug-in MicrobeJ (65). For batch culture experiments (overexpression and deletion mutants), the significance of the difference in cell size (length and/or area) between mutant versus parent strain cells was calculated using Welch’s modified *t* tests on ordered-quantile-normalized data, and the effect sizes of these differences were calculated using Cohen’s D test. For aphidicolin experiments, mixed-effects ANOVA with Welch’s *post hoc t* tests on ordered-quantile-normalized data were used to determine statistical significance of the differences between strains and time points. In all cases, *P* values were adjusted for multiple hypothesis testing using the Benjamini-Hochberg correction.

### Single-cell microfluidics time-lapse microscopy.

***(i) Strains and growth conditions.*** All strains analyzed in the mother machine experiments were streaked fresh from –80°C stocks and inoculated in 50 ml of CM supplemented with uracil in beveled flasks and grown at 42°C to an OD_600_ of 0.6 to 0.8 before loading into the microfluidic chip.

***(ii) Microfluidic chip fabrication.*** Microfabrication of the master mold used to create the microfluidic chip was performed in our mother machine experiments as previously described (45, 66), except that the second, wider layer in the cell chambers meant to enhance growth was not necessary to maintain the cell’s growth over the timescale of the current experiment. Specifically, to fabricate the microfluidic device, the features were molded into a piece of polydimethylsiloxane (PDMS) by pouring dimethyl siloxane monomer (Dow Sylgard 184 silicone elastomer base) mixed with a curing agent (Dow Sylgard 184 silicone curing agent) in a 10:1 ratio on top of the master mold, followed by degassing under a vacuum, and curing the setup overnight at 65°C. The solidified PDMS piece was then peeled from the master and cut into approximately 1.5 × 1.5-cm chips. Access holes for each of the feeding channels were punched using a 0.75-mm biopsy punch (WPI). The PDMS chip was then bonded to a KOH-cleaned 22 × 60-mm glass coverslip (VWR, no. 1.5) by oxygen plasma treatment at 200 mTorr of pressure and 30 W for 30 s in a PE-50 compact benchtop plasma cleaning system (Plasma Etch). Chips were baked at 65°C for at least 1 h before use.

***(iii) Loading Hbt. salinarum cells into microfluidic devices.*** CM medium supplemented with 1% bovine serum albumin was manually injected into the microfluidic device using a 1-ml syringe and incubated for 1 h. To avoid the crystallization of salt from the media, microcapillary pipet tips with CM media were left attached to the access holes of the chip. Prior to loading, cell cultures were filtered through a 40-μm cell strainer (EASYstrainer; Greiner Bio-One) to remove large salt crystals formed during culture growth. Cultures were then centrifuged at 3,000 × *g* for 5 min and concentrated to a final volume of 100 μl. Cells were manually loaded into the main flow channel of the microfluidic mother machine device using a 1-ml syringe. To increase the number mother machine wells containing cells, the chip was briefly centrifuged in a VWR Galaxy minicentrifuge. The microfluidic chip was then connected to automatic Harvard Apparatus syringe pumps by Tygon tubing (Saint-Gobain, ID 0.020 in) and blunt end dispense tips (Fisnar, 21 gauge, 1 in.). Fresh medium was continuously pumped at 1 to 2 μl/min at 37°C for 30 min to allow cells to further propagate within the mother machine wells. The system was subsequently moved to the microscope for data collection.

***(iv) Microscopy.*** Cells in the mother machine were imaged in a Nikon Eclipse Ti microscope with a 6.5-μm pixel CMOS Hamamatsu camera and a Nikon 100× NA 1.45 phase-contrast objective. Images were captured every 20 min for 1 to 2 days. Exposure times for both phase-contrast and fluorescence were 200 ms. Epi-illumination was provided by a fiber coupled Agilent launch for 488 nm to image msfGFP-FtsZ1 in strains AKS137, AKS170, and AKS196.

***(v) Single-cell growth analysis from the mother machine.*** Using phase-contrast images, 80 Δ*ura3*, 108 Δ*cdrS*, and 110 Δ*ftsZ2* cells were manually traced in Fiji image analysis software (63) to determine the cell area through pixel counting. These measurements were used to determine the cell area doubling (elongation) rates by fitting an exponential curve to the single-cell growth rate graphs of change of the cell area over time. *Hbt. salinarum* has previously been shown to grow exponentially through single-cell analysis (27). The area at birth was calculated from images immediately after complete the separation of daughter cells. The area during division was determined by adding the area of the two daughter cells immediately after division. The division site placement was determined by treating each pole separately and then calculating the ratio of each daughter cell to its corresponding parent. Single-cell measurements shown in the figures were plotted using ggplot2 (67) in the RStudio coding environment. Statistical analyses were conducted using the sjstats (68) and rstatix packages in RStudio. Significance of differences in doubling time between strains were modeled using 3-way ANOVA, with effect sizes calculated using an η^2^ test. The significances of the differences between mother or daughter cells in parent and mutant strains were calculated using Welch’s modified *t* tests on ordered-quantile-normalized data and the effect size of these differences calculated using Cohen’s D test. *P* values were adjusted for multiple hypothesis testing using the Benjamini-Hochberg correction.

### Gene expression analysis with NanoString.

To collect RNA across the growth curve, culture aliquots were collected from flask cultures at 4 phases of growth (early logarithmic, mid-logarithmic, early stationary, and late stationary phases). A 50-ml portion of culture was sampled at a low OD_600_ (∼0.05), followed by 2 ml at a higher OD_600_ (∼2.0). RNA was collected after 6 h of aphidicolin treatment and 11 h after release. Culture samples were centrifuged at 21,000 × *g* for 2 min; the supernatant was removed and immediately frozen in liquid nitrogen. Pellets were stored at −80°C overnight, and RNA was purified using an Absolutely RNA Miniprep kit per manufacturer’s instructions (Agilent, Santa Clara, CA). To verify the lack of DNA contamination, endpoint PCR was conducted for 30 cycles on 200 ng of RNA sample using the primers indicated in Table S3 in the supplemental material. RNA quality was determined using a bionanalyzer and RNA Nano 6000 chip according to the manufacturer’s instructions (Agilent).

Gene expression was quantified using NanoString detection and a custom probe Codeset (see Table S7 in the supplemental material) (69). Probes were designed to target 20 genes predicted to encode proteins involved in cytoskeletal and growth functions, and 3 control genes. One hundred nanograms of RNA was hybridized and quantified using the nCounter instrument by the Duke Microbiome Shared Resource core facility. Counts were normalized using three housekeeping genes (*eif1a2* [*VNG_RS06805*], *coxA2* [*VNG_RS02595*], and *VNG1065C* [*VNG_RS04150*]) and NanoString nSolver software and then further normalized to expression in the NRC-1 wild-type control. The significance of this relative normalized differential expression between the parent and Δ*cdrS* strain was assessed using the maSigPro package (70) in the R Bioconductor coding environment with the default parameters (except a Q-value of 0.01, alfa = 0.01, and an *R*^2^ cutoff of 0.7). We conducted Benjamini-Hochberg correction for multiple hypothesis testing in the context of the maSigPro package. All raw and normalized data and probe sequences are available in Table S7 in the supplemental material. The R code is presented in the github repository associated with this study (https://github.com/amyschmid/cdr).

### ChIP-seq experiment and analysis.

Triplicate cultures of the AKS113 (CdrL tagged at the C terminus with the FLAG epitope; see Table S2) and Δ*ura3* control strains were grown until stationary phase and subcultured in rich media supplemented with uracil. At mid-log phase (OD_600_ ∼0.15) and early stationary phase (OD_600_ ∼1.8), cultures were cross-linked and immunoprecipitated as described previously (59), with the following exceptions: (i) cultures were cross-linked with 1% formaldehyde for 30 min at room temperature, and (ii) immunoprecipitations were conducted using Dynabead magnetic beads (Thermo-Fisher product 10002D) conjugated with anti-FLAG (Abcam ab1162) anti-rabbit monoclonal antibody at a 1:250 dilution. DNA concentration was determined by using a NanoDrop (Thermo Scientific). Libraries were constructed using the KAPA Hyper Prep kit and Illumina TruSeq adapters. DNA library quality was assessed by bioanalyzer using a high-sensitivity DNA chip (Agilent). Samples were pooled and run in a single lane on an Illumina HiSeq 4000 (Duke Sequencing and Genomics Technologies core). Next, 50-bp single reads were assessed for quality using FastQC (www.bioinformatics.babraham.ac.uk), and adapter sequences were trimmed using Trim Galore (www.bioinformatics.babraham.ac.uk) and Cutadapt (71). Resultant sequences were aligned to *H. salinarum* NRC-1 genome (RefSeq NC_002607.1, NC_002608.1, and NC_001869.1) using Bowtie2 (72). Subsequent analyses were conducted in the R Bioconductor coding environment, and all associated code is freely available (https://github.com/amyschmid/cdr). Peaks were called using MOSAiCS (73) from sorted bam files with the following arguments: fragment length, 200; bin size, 200; read capping, 0; analysis type, IO; background estimate, rMOM, signal model 2S; and FDR, 0.01. Peaks reproducible across two of three biological replicate samples were integrated using the DiffBind (74) and ChIPQC (75) packages. Peak locations were associated with annotated genes using the IRanges Bioconductor package (76). Data were visualized for the figures using the R package trackViewer (77). R package version numbers are given in the github repository (https://github.com/amyschmid/cdr). Raw and analyzed data are available through GEO accession GSE148065.

### Data availability.

All gene expression and ChIP-seq data from this study are available to the public through GEO accession GSE148065. Whole-genome resequencing data are available via Sequence Read Archive Project PRJNA614648. Code and data sets are available on the GitHub repository https://github.com/amyschmid/cdr. All supplementary figures, tables, and movies are available from FigShare at https://doi.org/10.6084/m9.figshare.12195081.
